# Capture of high numbers of *Simulium* vectors can be achieved with Host Decoy Traps to support data acquisition in the onchocerciasis elimination endgame

**DOI:** 10.1016/j.actatropica.2021.106020

**Published:** 2021-09

**Authors:** Blaise Armand Defo Talom, Peter Enyong, Robert A. Cheke, Rousseau Djouaka, Frances M. Hawkes

**Affiliations:** a University of Dschang, Cameroon; b Research Foundation for Tropical Diseases and Environment, Cameroon; c Natural Resources Institute, University of Greenwich, United Kingdom; d International Institute of Tropical Agriculture, Benin

**Keywords:** Onchocerciasis, *Simulium*, Vector surveillance, Host Decoy Trap, Elimination

## Abstract

Onchocerciasis elimination is within reach in many countries but requires enhanced surveillance of the *Simulium* vectors of *Onchocerca volvulus*. Collection of sufficient numbers of adult *Simulium* to detect infective *O. volvulus* larvae is hindered by limited sampling tools for these flies. Here, we tested for the first time the Host Decoy Trap (HDT), an exposure free method previously developed for *Anopheles* vectors of malaria parasites, as a potential sampling tool for adult *Simulium*. In three replicates of a randomized Latin square experimental design, the HDT was compared to Human Landing Catches (HLC) and the Esperanza Window Trap (EWT). A total of 8,531 adult *S. damnosum* sensu lato blackflies (*S. squamosum* group) were found in catches from the three different trapping methods. The HDT (mean catch 533 ± 111) caught significantly more *S. squamosum* than the EWT (mean catch 9.1 ± 2.2), a nearly 60-fold difference. There was no significant difference between the HLC (mean catch 385.6 ± 80.9) and the HDT. Larvae indistinguishable from those of *O. volvulus* were dissected from 2.86% of HDT samples (*n* = 70) and 0.35% of HLC samples (*n* = 285); a single infective third-stage larvae (L3) was  found during dissection of a sample from the HDT. Owing to its very high capture rate, which was comparable to the HLC and significantly greater than EWT, alongside the presence of infected flies in its catch, the HDT represents a potentially valuable new tool for blackfly collection in elimination settings, where thousands of flies are needed for parasite screening.

## Highlights

• Vector surveillance will be critical in the move towards onchocerciasis elimination.
• Host Decoy Traps (HDTs) caught 60 times more *Simulium* than esperanza window traps.
• A single infectious *Onchocerca* species larva was dissected from a sample caught by HDT.
• HDTs may support onchocerciasis surveillance in elimination campaigns.

## Graphical abstract

Host Decoy Traps are a potential surveillance tool for *Simulium* vectors in onchocerciasis elimination campaigns.

**Figure:**
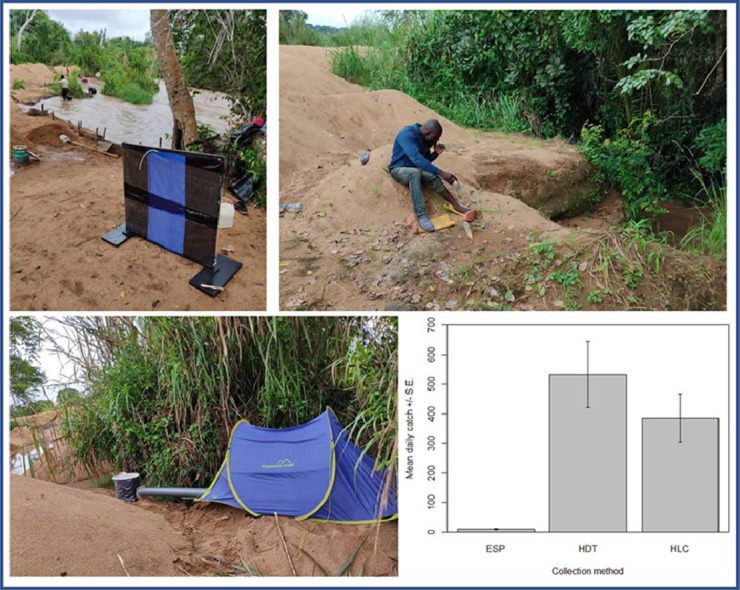

## 1 Introduction

Onchocerciasis is a neglected tropical disease caused by the filarial nematode *Onchocerca volvulus*, which live in the lymphatic and subcutaneous tissues of humans and can cause blindness. It can also lead to skin lesions and itchiness and is a major risk factor for epilepsy (Murdoch et al., 2002; Korevaar and Visser, 2013). The nematodes are transmitted by bites of infected blackflies belonging to the genus *Simulium* (Diptera: Simuliidae).

Onchocerciasis control in Africa has been achieved by vector control alone (McMahon et al., 1958; Traore et al. 2009; Garms et al., 2009), by mass drug administration (MDA) with the microfilaricide ivermectin (Katabarwa et al., 2020) or by combinations of both (Lakwo et al., 2017). However, onchocerciasis remains as a public health problem in many parts of Africa so MDA alone, or in combination with localized vector control, is underway in an attempt to reach the goal of continent-wide elimination (World Health Organization, 2020). Regardless of the control measure(s) involved, cessation of transmission can only be confirmed by examining samples of adult female vectors; a minimum of 6000 are needed for testing for the presence of *O. volvulus* infective larvae with the pool-screening method in a single transmission zone (A geographical area where transmission of *O. volvulus* occurs by locally breeding vectors and which can be regarded as a natural ecological and epidemiological unit for interventions) (World Health Organization, 2016). Therefore, a means to catch thousands of vector blackflies is still needed, especially a method that avoids the ethically problematic use of human landing catches (HLCs). Although the HLC is the gold standard method of estimating human-biting rate and intensity of transmission, it is plagued by ethical limitations and is laborious and increasingly expensive to implement.

Early research to understand which features might attract adult blackflies includes the work of Bellec (1974), who noted that black clothing resulted in increased catches of members of the *Simulium damnosum* complex and, given the attractive properties of carbon dioxide to the species group (Thompson 1976), he postulated that combining these cues might provide a trap at least sufficient to complement, if not replace, human baited collections. The use of traps to catch adult blackflies was reviewed by Service (1977) and, more recently, Rodriguez- Perez et al. (2013) developed the Esperanza Window trap (EWT) for blackfly sampling in Southern Mexico. The trap utilized a combination of olfactory cues and visual attractants to lure adult host-seeking blackflies and was found to collect *S. ochraceum* sensu lato, a dominant *Simulium* vector in Latin America, in similar numbers to an HLC. Toe et al. (2014) adapted and optimized a version of the EWT for African blackflies in Burkina Faso, focusing on the collection of *S. damnosum* sensu stricto (s.str.) and *S. sirbanum*. The results showed that this version caught a similar number or, on a few occasions, more blackflies than the HLC.

The principle of using attractive odours, particularly carbon dioxide and skin volatiles, has been established across host-seeking behaviours in many haematophagous and disease vector species groups, including mosquitoes (Diptera: Culicidae; Zwiebel and Takken, 2011), sandflies (Diptera: Psychodidae; Pinto et al., 2012) and assassin bugs (Hemiptera: Reduviidae; Guerenstein and Guerin, 2001), as well as blackflies (Thompson 1976, 1977). Attraction to several modalities of host-associated stimuli, including not only olfactory but also visual and physical stimuli, is likely to be conserved across haematophagous insect groups, as differentiation of hosts from non-hosts will rely on these specific stimuli.

Recently developed for mosquitoes of the *Anopheles gambiae* complex, and based on their behavioural responses to different host-associated stimuli, the Host Decoy Trap (HDT) (Hawkes et al., 2017) combines olfactory, visual and thermal cues to induce the full range of host-seeking behaviour in vectors, from long range activation and attraction, to close range orientation, approach and landing stimuli (Hawkes and Gibson, 2016; Spitzen et al., 2013), for the purposes of attracting and trapping mosquitoes. Briefly, the HDT mimics a potential human host by combining olfactory cues from a person in a tent, and thus protected from potentially infectious vectors, with high contrast visual stimuli and a thermal signal equivalent to human body temperature. These cues mimic a blood host and insects land on the surface of the trap, where they are stuck to a strong adhesive.

Previous trials with HDTs have focused on mosquito vectors of malaria (Abong'o et al., 2018; Davidson et al., 2020) and arboviruses (Tang et al., 2020), but other hematophagous vectors, including *Simulium* species, have been noted in trap catches (authors’ unpublished data). An advantage of this approach is that it has been designed for sampling in outdoor environments, so may be suitable for collection of *Simulium*, whose day-biting habits lead to much human-biting outdoors. Given the imperative for enhanced vector surveillance in areas transitioning towards onchocerciasis elimination, we therefore sought to establish whether the HDT might provide an alternative method of sampling *Simulium* vectors and undertook to compare this method to both EWTs and HLCs in an area of ongoing *O. volvulus* transmission in central Cameroon.

## 2 Materials and methods

### 2.1 Study site

Field research took place on the banks of the Mbam River in Bafia sub-division of the Centre Region, Cameroon (N 4°45′00′', E 11°17′47.288′'), around 120 km north of the capital Yaoundé. The Mbam River is a large, perennially flowing river, with multiple series of rapids close to Bafia that provide suitable conditions for blackfly larval development. The river's flow rate is influenced by the regular release of water from a retention dam, located upstream from Bafia in Magba. Biting by adult blackflies occurs throughout the year, with seasonal peaks occurring between February and May and between August and November. Bafia is one of five hyperendemic onchocerciasis foci in Cameroon, with disease prevalence above 60% (Tekle et al., 2016), and members of the *S. damnosum* complex are the only significant vectors of the parasite in this country (Traoré-Lamizana et al., 2001; Adler, 2021).

Onchocerciasis control is faltering in some parts of Cameroon. Only 3 of 11 health districts were close to elimination after 15 years of treatment with ivermectin (Katabarwa et al., 2013), there has been continuing transmission in the southwest after 10 years of ivermectin distribution (Wanji et al., 2015a) and compliance with programmes distributing the drug is low in some areas (Wanji et al., 2015b). Similar results with unacceptable levels of onchocerciasis persisting have also been reported from a variety of areas in the country (Eisenbarth et al., 2016; Kamga et al., 2016), although successes have also been documented (Kamga et al., 2017).

Three sampling points, at least 50 m apart, were located close to the rapids around 300 m upstream from a ferry crossing linking Bafia and Ngoro sub-divisions. Collections were carried out following a 3 x 3 x 3 randomised Latin square experimental design of traps x days x sampling points, over nine days, giving three Latin square replicates. Experiments took place over six hours each day, in two three-hour slots, the first between 08:00 and 11:00, and the second between 13:00 and 16:00, to provide collectors with a break during the day. All data were collected between October and November 2020.

### 2.2 Host Decoy Trap (HDT)

**Fig. 1:**
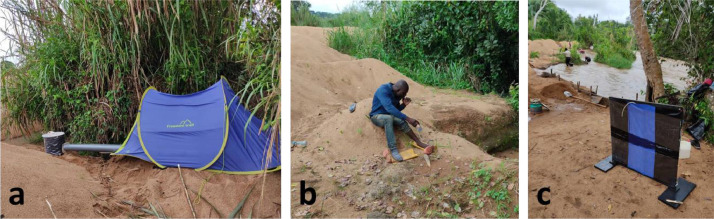
Three sampling methods for collecting blackflies; a: Host Decoy Trap, b: Human Landing Catch, and c: Esperanza Window Trap.

The HDTs (Biogents GmbH, Germany) were prepared as previously described (Abong'O et al., 2018) (Fig. 1a). Briefly, the trap provides olfactory, thermal and visual stimuli to attract blood-seeking insects. The olfactory cue is derived from a person resting in a tent. A 12 V fan inside the tent is connected to a length of ~10 cm diameter pipe, which draws the naturally occurring body odours and exhaled carbon dioxide from the tent to the base of a barrel-shaped trap. Netting over the end of the pipe prevents insects entering the tent. The visual cue is provided by fitting the barrel-shaped trap with a plain black cloth cover to provide high visual contrast against the surrounding ground. The thermal cue is provided by filling the trap with warm water; the trap is insulated so as to maintain human body temperature (35±5 °C) for a minimum of 12 h. A clear adhesive plastic sheet (Barrettine Environmental Health, UK) is wrapped around the surface of the trap to catch host-seeking haematophagous insects as they land. Full instructions for constructing an HDT from scratch are available online (Hawkes et al., 2018), and a user-guide is provided with the product sold by Biogents.

### 2.3 Human landing catch (HLC)

The HLC was performed as follows. A human volunteer sat at a collection site with his legs exposed and collected any blackfly landing on his legs before it took a blood meal (Fig. 1b). Flies were collected using a mouth aspirator or single 5 ml tubes. In the latter case, the flies were kept secure in the tube by a ball of cotton wool.

### 2.4 Esperanza Window Trap (EWT)

The EWT version B was used in this study (Toé et al., 2014). It consisted of a 1 m^2^ target of black and blue satin fabric fixed on a wooden frame by pins (Fig. 1c). The surfaces of the fabric were coated with Tangle Trap™ insect trap coating paste (Contech, Victoria, BC, Canada) to trap landing insects. Carbon dioxide was used as the attractive odor and generated through yeast fermentation using 17.5 g dried yeast, 250 g sugar and 2.5 l water in a 5 l plastic container (Smallegange et al., 2010). The carbon dioxide was directed onto the vertical surface of the adhesive fabric via a plastic tube fixed into the plastic container.

### 2.5 Species identification

Adult female blackflies were identified morphologically as *S. damnosum* s.l. according to the key of Freeman & de Meillon (1953) and to the *S. squamosum* group of the *S. damnosum* complex by thorax: antenna ratios (Garms and Cheke 1985). The samples were not identified to a particular cytoform but previously *S. squamosum* A and *S. mengense* have been found in  the Mbam river (Barbazan et al., 1998; Traore-Lamizana et al., 2001), as well as *S. squamosum* cytoform E2 (Hendy 2018).

### 2.6 Parasite detection

A sub-sample of collected blackflies was dissected for the presence of *Onchocerca* larvae. Flies collected by HLC were anesthetized using chloroform and dissected while those stuck to the adhesive of the HDT, generally by the legs, wings and abdomen, were carefully removed using fine forceps before dissection. The abdomen was opened to determine the parity while the head and thorax (for the parous flies) were dissected and any *Onchocerca* larvae (L1, L2, L3) indistinguishable from those of *O. volvulus* identified morphologically whenever present (WHO, 2002), but the possibility of them being the cattle parasite *O. ochengi* could not be ruled out.

### 2.7 Statistical analysis

Data were analysed in R version 3.5.2 (R Core Development Team, 2018) using the packages *MASS* (Venables and Ripley, 2002) and *multcomp* (Hothorn et al., 2008). Generalised linear models were produced using a Poisson distribution and negative binomial distribution, the latter giving the better fit according to residual deviance and Akaike Information Criterion. *Post hoc* Tukey contrasts were used to make comparisons between trapping methods.

## 3 Results

**Fig. 2:**
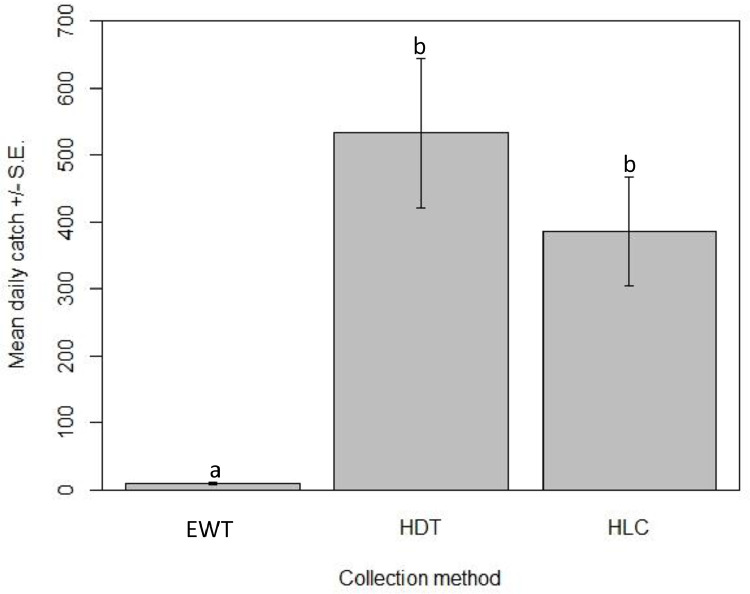
Comparison of three collection methods (mean daily catch ± standard error). Different letters denote significant differences at *P* < 0.01 by negative binomial generalised linear models. EWT: Esperanza Window Trap; HDT: Host Decoy Trap; HLC: Human Landing Catch.

A total of 8351 blackflies  (*S. squamosum* sensu lato) were collected from three trap methods. The HDT collected the greatest number of flies (*n* = 4799, 57%), followed by HLC (*n* = 3470, 42%) and EWT (*n* = 82, <1%). Trapping method was a significant factor determining catch (*z* = 9.4, *P* < 0.001). Significantly more *S. squamosum* were collected with the HDT (mean catch 533.2 ± 111.7, *z* = 12.9, *P* < 0.001) and the HLC (mean catch 385.6 ± 80.9, *z* = 11.9, *P* < 0.001) than with the EWT (mean catch 9.1 ± 2.2) (Fig. 2). While more blackflies were collected by the HDT overall, there was no significant difference between the mean catch of the HDT and the HLC (*z* = −1.1, *P* = 0.517).

**Fig. 3:**
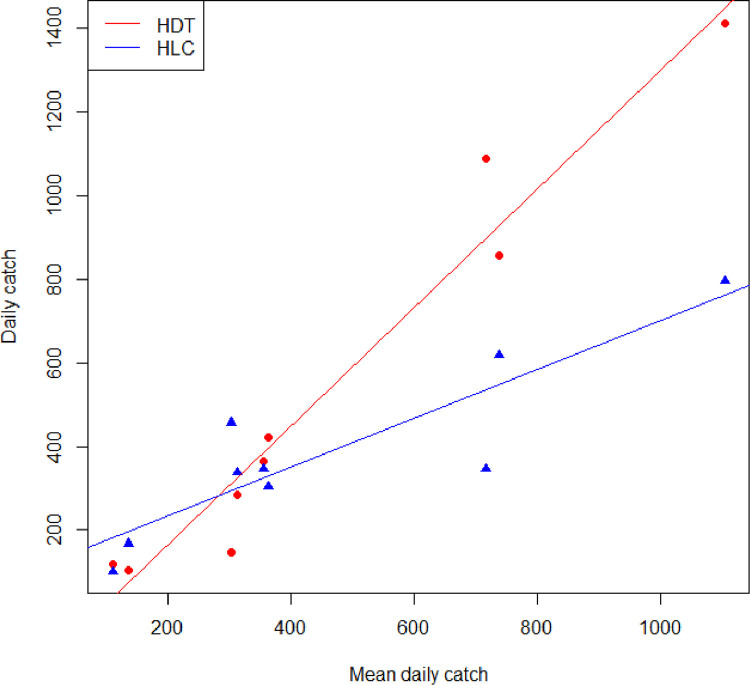
Daily catch of *S. squamosum* by HDT (red circles) and HLC (blue triangles) against mean daily catch (HDT+HLC/2).

To investigate the relationship between HDT and HLC collections, and because this is a relatively small data set, these can be related descriptively to the mean of their catch (Fig. 3). There is relatively good accord between the HLC and HDT when mean catches are below 400 blackflies per day. However, at higher mean daily densities, the HDT tended to collect more than the HLC, suggesting a potential density dependent effect that requires further sampling to investigate.

Of the 355 specimens dissected, larvae of *O. volvulus* were found in three specimens. These were found in both the HDT and HLC (Table 1), although an infective stage (L3) was only dissected from one sample from the HDT.

**Table 1:** Results from dissections for larvae indistinguishable from *O. volvulus* in samples of *S. squamosum* from two different collection methods. HDT: Host Decoy Trap; HLC: Human Landing Catch.

Collection method	No. of flies dissected	No. of positive flies	% of *Onchocerca* larvae	Larval stage		
				L1	L2	L3
HDT	70	2	2.86%	9	0	1
HLC	285	1	0.35%	0	4	0

**Fig. 4:**
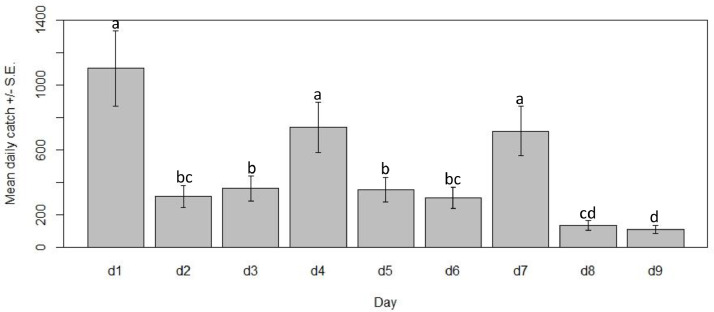
Mean catch of *S. squamosum* derived from both Host Decoy Trap and Human Landing Catches. Different letters denote significant differences at *P* < 0.01 by negative binomial generalised linear models. (For interpretation of the references to colour in this figure legend, the reader is referred to the web version of this article.)

When analyzing the effect of day, the EWT was excluded because of its low catches, which represented less than 1% of all specimens collected. Focusing on the HDT and HLC, then, over the course of data collection, there appeared to be a downward trend in mean catch from the beginning to the end of the experiment. The sampling duration was relatively brief (nine consecutive days) and there were typical fluctuations in mean daily catches derived from the HDT and HLC as might be expected due to natural variations in the adult population (Fig. 4), with Days 1, 4 and 7 having significantly larger mean collections than any other days (*P* < 0.01). However, significant differences between daily mean catch were found between Day 9, the last day of collection, and every other experimental day (*P* < 0.01) except the immediately preceding day, Day 8 (*P* = 0.99).

## 4 Discussion

In this paper, we present the first trial of HDTs for trapping *Simulium* vectors. In comparison to the EWT, the HDT vastly outperformed this method in terms of overall catch, collecting nearly 60 times as many *S. squamosum* over the study period. There was good accord between the HLC and HDT, with the HDT offering the added benefit that the collector is protected from the bites of not only blackflies but all other hematophagous insects that may be present, including other potential disease vectors. In addition, there is preliminary evidence that the HDT collects larger numbers of blackflies when the density of flies is greater. Thus, the HDT may be well-suited to sampling blackflies in areas of high biting pressure, at least in this local context, while remaining sensitive at lower densities. Surveillance of onchocerciasis using the pool-screen method requires 6000 flies for detection of infective *O. volvulus* larvae (World Health Organization, 2016), which in the context of the current study could be feasibly achieved by using one HDT every day for less than two weeks; in comparison it would take over a year and half to collect sufficient blackflies using an EWT for the pool-screen method to be used. The HDT could therefore be a valuable tool in elimination surveillance.

The performance of different sampling methods can often vary between location and even between closely related species. In Tanzania, EWTs did not perform well relative to HLC for the collection of *S. damnosum* s.l., while the same study protocol followed in Uganda also showed poor performance for the anthropophilic *S. bovis* but results comparable to an HLC for *S. damnosum* s.l. (Hendy et al., 2017), highlighting the importance of local testing and calibration. Also, our results with low catches on EWTs contrast with those of Toé et al. (2014), who found that EWTs were as good as human vector collectors in terms of trapping the savannah species *S. damnosum* s. str and *S. sirbanum.* However, our experiments were conducted in a forest region trapping *S. squamosum* and this may explain the differences since savannah members of the *S. damnosum* complex use visual cues when host-seeking more than forest species that tend to use odor cues (Thompson 1976, 1977). The variation in mean daily catch could be attributed to a number of factors, including changing environmental conditions, although we did not record environmental variables during the period of data collection and cannot draw any conclusions regarding this.

Previous research with *Anopheles* mosquitoes showed that it is possible to run PCRs for *Plasmodium* detection and sibling species identification using samples collected by and extracted from HDTs (Hawkes et al., 2017). Owing to supply chain issues arising from the SARS-CoV-2 pandemic, it was not possible to obtain the reagents required for molecular determination of *O. volvulus* in the collected blackfly samples. Instead, dissections were performed. These were challenging with samples obtained from the HDT, as the solvent used to remove samples caused them to become dry and brittle. However, it was found to be possible to dissect the flies while they remained *in situ* on the adhesive sheet, demonstrating this established technique is possible on HDT samples, although molecular detection would be more practical, and its feasibility should be confirmed.

Recent work has sought to assess the suitability of EWTs as a potential vector control tool against *S. damnosum* in Uganda. It was found to significantly reduce biting rates in one location, but to make no difference when deployed in a second area (Loum et al., 2019). The use of attractive traps to lure and kill adult blackflies warrants further investigation and local testing in areas with high human-biting rates. The HDT represents one such potential method for controlling adult blackflies due to the large numbers collected, and may be suited to the situation in Cameroon, especially as blackflies collected via the HDT included samples that were positive for *Onchocerca* larvae. This would be a valuable complement to MDA and larval control in areas of persistent infection.

In conclusion, the HDT represents a potentially powerful means of collecting the large number of blackflies required for surveillance during onchocerciasis elimination campaigns. There is also potential for the tool to be applied as a form of adult vector control against *Simulium*.

## Authorship statement

**Blaise Armand Defo Talom:** Investigation, Writing – review & editing. **Peter Enyong:** Investigation, Writing – original draft. **Robert A. Cheke:** Conceptualization, Writing – review & editing, Funding acquisition. **Rousseau Djouaka:** Conceptualization, Writing – review & editing, Project administration, Funding acquisition. **Frances M. Hawkes:** Conceptualization, Formal analysis, Writing – original draft, Project administration, Funding acquisition.

## Funding

We are grateful to the Biotechnology and Biological Sciences Research Council (BBSRC) for funding this project through the Gnatwork pump-prime funding, reference BB/R005362/1.

## Ethical approval

This research was reviewed and approved by the International Institute of Tropical Agriculture's Internal Review Board and received ethical clearance reference: IITA-IRB/HDT-surveillance-disease-vectors.

## Declaration of Competing Interest

The authors declare that they have no known competing financial interests or personal relationships that could have appeared to influence the work reported in this paper.
